# The Use of Rhinomanometry in Mouth Breathing: A Systematic Review of the Literature *

**DOI:** 10.1055/s-0044-1785199

**Published:** 2024-03-27

**Authors:** Merly Fernanda Illera Castellanos, Hilton Justino da Silva, Silvio Ricardo Couto de Moura, Luciana de Barros Correia Fontes, Niedje Siqueira de Lima, Thiago Freire Pinto Bezerra, Daniele Andrade da Cunha

**Affiliations:** 1 Human Communication Health Post-Graduate Program, Universidade Federal de Pernambuco, Recife, PE, Brazil; 2 Department of Speech Therapy, Centro de Ciências da Saúde, Universidade Federal de Pernambuco, Recife, PE, Brazil; 3 Department of Dentistry, Centro de Ciências da Saúde, Universidade Federal de Pernambuco, Recife, PE, Brazil; 4 Department of Otorhinolaryngology, Hospital das Clínicas, Universidade Federal de Pernambuco, Recife, PE, Brazil

**Keywords:** rhinomanometry, airway resistance, mouth breathing

## Abstract

**Introduction** Mouth breathing generates imbalances in the musculature, in craniofacial morphofunctionality, and in the stomatognathic system. Therefore, it is essential to make a diagnosis of mouth breathing through the quantitative assessment of nasal permeability, which can be performed through rhinomanometry.

**Objective** To investigate the effectiveness of rhinomanometry in the diagnosis of mouth breathing in pediatric patients through a systematic review of the literature.

**Data synthesis** The guiding question was: “Is the use of rhinomanometry as an assessment tool effective in the diagnosis of mouth breathing in pediatric patients?”. We conducted a search on the following databases: Latin American and Caribbean Center on Health Sciences Information (BIREME), Latin American and Caribbean Health Sciences Literature (LILACS), PubMed/Medical Literature Analysis and Retrieval System Online (MEDLINE), Scientific Electronic Library Online (SciELO), Web of Science, and Science Direct. The Health Sciences Descriptors (Descritores em Ciências da Saúde, DECS, in Portuguese) and Medical Subjects Headings (MESH) were combined with the Boolean operator AND in the search strategy: *rhinomanometry* AND *mouth breathing* AND *diagnosis* AND *nasal pressure* AND *nasal airflow* AND *nasal resistance* . Observational cohort and cross-sectional studies that addressed the effectiveness of rhinomanometry in the diagnosis of mouth breathing were included. The reviewers independently extracted the information and scored the review quality based on the Physiotherapy Evidence Database (PEDro) scale and the grading of evidence levels according to the Grading of Recommendations Assessment, Development and Evaluation (GRADE) system. Of the 1,536 articles identified, only 3 were selected for the present review after the application of the eligibility criteria.

**Conclusion** There is great concern regarding the assessment of nasal function. There was a lack of standardization of rhinomanometry to test the effectiveness of nasal resistance as an aid in the diagnosis of breathing mode.

## Introduction

Nasal breathing is the human physiological breathing, and it exerts a great influence on the organization of other orofacial functions. 1 When nasal breathing is partially (mixed breathing) or totally (oral breathing) replaced, there is a change in the individual's body organization, and its persistence is responsible for important muscle imbalances, with repercussions on craniofacial morphofunctionality and on the stomatognathic system. 2

The oral breathing pattern can occur when structural nasal obstructions, permanent or not, prevent the passage of air through the nose and, therefore, the person breathes through the mouth. There are complementary methods of airway assessment, in a multidisciplinary context, which help in the diagnosis of mouth breathing. Specific nasal permeability tests have been used for many years to quantify the complex symptom of nasal obstruction, guiding the therapeutic approach, such as the Glatzel and Altmann mirrors, and modified inspiratory or expiratory flow meters for nasal use.

However, one of the most recent methods to evaluate nasal function and obstruction is rhinomanometry, which enables the quantification of the transnasal airflow and pressure gradient, which, in turn, enables the calculation of nasal resistance during a respiratory cycle. 3 Currently, there are three rhinomanometry methods in use: anterior rhinomanometry, posterior (oral) rhinomanometry, and postnasal (pernasal) rhinomanometry. Active anterior rhinomanometry (AAR) evaluation is considered the most used modality to assess resistance, and it is often recommended for its easy technique.

Although rhinomanometry is a current instrument in the clinical practice of speech therapists, it is worth noting that this resource has been used by other health professionals since the 1930s. 4 5 6 Recently, with technological advances and with the growing interest of professionals in this subject, studies have been conducted on the applicability of new techniques and also regarding the use of microcomputers connected to measuring devices that try to quantify and objectively evaluate the respiratory function of the nasal airway. 7 8

The information obtained from the exam on the degree of nasal obstruction is useful to establish comparisons and demonstrate the effectiveness of decongestant therapies 5 and surgical procedures, 9 which comprise the management of disorders of the respiratory mode due to nasal obstruction. 10

Nowadays, the need for this method of assessment is evidenced due to changes in nasal respiratory physiology that manifest a relationship between the upper and lower airways. 11 Although rhinomanometry does not provide an etiological diagnosis of nasal obstruction, it is noteworthy that its application enables the quantification of the magnitude of the obstruction by measuring nasal resistance and the nasal cavities, thus favoring an assessment of nasal permeability. Therefore, the present article aims to investigate the effectiveness of rhinomanometry in the diagnosis of mouth breathing in pediatric patients through a systematic review of the literature.

## Review of the Literature

### Search Strategy

The present study sought to answer the guiding question: “Is the use of rhinomanometry as an assessment tool effective in the diagnosis of mouth breathing in pediatric patients?”. The present systematic review was carried out from March 2020 to July 2020 according to the Population, Intervention, Comparator, and Outcome (PICO) strategy ( Table 1 ).

**Table 1:** Eligibility criteria for the studies considered for the present review

Question: Is rhinomanometry as an assessment tool effective as a diagnostic aid in mouth breathing pediatric patients?		
Selection criteria	Inclusion criteria	Exclusion criteria
Population	Mouth breathing patients	Healthy individuals
Intervention	Rhinomanometry	The use of instruments other than rhinomanometry
Comparator	Comparison of the same instrument to assess nasal permeability	No comparison of the same instrument
Outcome	Effectiveness of rhinomanometry as an assessment tool for oral breathing diagnosis and identification of quantitative variables of nasal function, nasal flow, and resistance	Results of other instruments that assess nasal permeability
Type of study	Randomized clinical trials, cohort studies, case-control studies, and cross-sectional studies	Animal studies; ecological studies; opinion articles; review articles (of the literature, as well as systematic and narrative reviews); case reports; and theses

To select the terms used in the database search, we used free terms (FTs), which are those not found in the Medical Subjects Headings (MESH) but are relevant for research, as well as the MESH, which are obtained from an international data search platform. The following terms were used: *Rhinomanometry* (MESH) OR *Nasal Airflow* (FT) OR *Nasal Resistance* (FT) AND *Mouth Breathing* (MESH) OR *Breathing, Mouth* (MESH) OR *Mouth Breathings* (MESH), and their possible combinations in Portuguese, English, and Spanish.

The search was performed in the following databases: Latin American and Caribbean Center on Health Sciences Information (BIREME), Latin American and Caribbean Health Sciences Literature (LILACS), PubMed/Medical Literature Analysis and Retrieval System Online (MEDLINE), Scientific Electronic Library Online (SciELO), Web of Science, and Science Direct. The present review was registered on the International Prospective Register of Systematic Reviews (PROSPERO, under identification number CRD42020204677).

The inclusion criteria were: cross-sectional original articles, case-control studies, cohort studies, and randomized clinical trials, which addressed the effectiveness of rhinomanometry in the diagnosis of mouth breathing in pediatric patients. The exclusion criteria were: animal studies, ecological studies, opinion articles, review articles (of the literature, systematic, and narrative), case reports, and theses, as well as articles that did not mention the topic addressed in the present review and that did not use rhinomanometry to assess oral breathing.

According to the eligibility criteria, two independent evaluators preselected the articles by title and abstract. Then, the full text of the preselected articles was read to assess if they had performed a descriptive analysis of the effectiveness of rhinomanometry in the diagnosis of mouth breathing. If there was disagreement between the reviewers, a third researcher would be consulted to reach a consensus.

### Data Analysis

For the preselection of studies, the titles and abstracts of all publications were rigorously read according to the inclusion criteria. In the cases in which the title and abstract were not sufficient to determine whether the article met the inclusion criteria, the publication was analyzed in its entirety; after the preselection, the full text of each study was read. At this stage, meetings with the authors of the research were organized to clarify doubts regarding the inclusion or exclusion of studies. This procedure aimed to reduce the bias in the selection of studies, thus providing greater reliability.

The articles selected after the full-text reading were analyzed using a protocol that considered the following data: author, year of publication, country, type of study, population/sample, age of the patients, objective, materials and methods used, duration of the treatment, and main results ( Table 2 ).

**Table 2:** Summary of the studies included for analysis

Author, year	Country	Type of study	Sample	Objective	Materials and methods	Main results
Itikawa et al., 2012 14	Brazil	Cohort study	29 patients with mouth breathing with posterior crossbite of both genders, aged between 7 and 10 years. Orthodontic and otorhinolaryngological documentation was performed at three different times: before expansion, immediately after ,and 90 days after expansion.	To evaluate the effect of rapid maxillary expansion on the nasal cavity and facial morphology through acoustic rhinometry and computed rhinomanometry.	Acoustic rhinometry (ARM) and rhinomanometry (RMM). The SR2000 equipment, (Rhinometrics A/S, Smørum, Hovedstaden, Denmark). Complete orthodontic documentation (lateral and posteroanterior cephalometric radiographs, study models, extraoral frontal and profile photographs, and intraoral photographs). Rapid treatment of the maxilla (maxillary expansion).	Mean nasal resistance during inspiration (inspiration at T 3 = 2.231 lm /cmH2O) and expiration (expiration at T3 = 1,828 lm /cmH2 O) were significantly lower after treatment than before treatment (inspiration at T1 = 3,368 lm/cm H2O and expiration at T1 = 2,675 lm/cmH2O).
Sakai et al., 2018 15	Brazil	Cross-sectional study	30 mouth-breathers with maxillary atresia (age: 7–13 years) with posterior crossbite.	To evaluate the correlation between acoustic rhinometry, computed rhinomanometry, and cone-beam computed tomography in mouth breathers with maxillary atresia.	Acoustic rhinomanometry: nasal volumes and minimal cross-sectional areas of the nasal cavity; computed rhinomanometry: nasal flow and mean inspiratory and expiratory resistances; cone-beam computed tomography and administration before and after the vasoconstrictor.	Negative correlations were found: i) width of 4 and mean inspiratory resistance (Rho = − 0.385); ii) mean inspiratory resistance before vasoconstrictor administration and a volume of 0-5 cm (Rho = − 0.382); and iii) mean expiratory resistance before vasoconstrictor administration and minimum cross-sectional area 1 (Rho = − 0.362)
Ramos et al., 2019 16	Brazil	Prospective cohort study	59 children: 30 mouth-breathers with indication for adenotonsillectomy evaluated before and six months after surgery, and 29 nasal-breathers. Age between 2 and 12 years.	To correlate total inspiratory nasal airflow (TINAF) and pulmonary artery systolic pressure (PASP) in mouth-breathing children (MB) before and after adenotonsillectomy and in nasal breathers (NB).	Application of a questionnaire to the child's guardians to obtain information about upper airway obstruction and sleep-disordered breathing, according to the protocol proposed by Chervin et. al. Assessment of PASP through transthoracic echocardiography. Nasal permeability evaluation using anterior rhinomanometry to estimate nasal flow, pressure, and resistance. (TINAF)	Nasal flow in MBs was of 266.76 preoperatively and of 498.93 postoperatively. In NBs, it was of 609.37. The mean nasal patency in the preoperative period was of 42.85%, and of 79.33% in the postoperative period. Among NBs, it was of 112.94%.

### Study Quality Analysis and Data Extraction

The methodological quality of the selected studies was assessed using the Physiotherapy Evidence Database (PEDro) scale. 12 The choice of this scale was based on its detailing and scope of the methodological quality of research.

The methodological characteristics of the articles were analyzed according to the inclusion criteria, as well as the statistical analyses and statistical comparison of the selected groups in each study ( Table 3 ).

**Table 3:** Result quality on the Physiotherapy Evidence Database (PEDro) scale

	Study		
	Itikawa et al., 2012 14	Sakai et al., 2018 15	Ramos, et. al. 2019 16
1. Eligibility criteria were specified	1	1	1
2. Subjects were randomly allocated to groups (in a crossover study, subjects were randomly allocated in the order in which the treatments were received)	0	0	0
3. Allocation was concealed	0	0	?
4. The groups were similar at baseline with respect to the most important prognostic indicators	0	0	1
5. All subjects were blinded	0	0	0
6. All therapists who administered the therapies were blinded	0	0	0
7. All assessors who measured at least one key outcome were blinded	0	0	0
8. Measures of at least 1 key outcome were obtained from > 85% of the subjects initially allocated to groups	1	1	1
9. All subjects to whom the outcome measures were available received the treatment or control condition as allocated or, when this was not the case, data for at least one key outcome was analyzed by “intention-to-treat”.	1	1	1
10. The results of between-group statistical comparisons were reported for at least one key outcome	1	1	1
11. The study provides both point measures and measures of variability for at least one key outcome	1	1	1
Total	5	5	6

### Risk of Bias in Individual Studies

The studies included were independently assessed for risk of bias using the Grading of Recommendations Assessment, Development and Evaluation (GRADE) system 13 for each important finding in each review.

Objective criteria were used to assess the quality of evidence for each outcome in the following GRADE domains: methodological limitations (risk of bias); study design; study quality; inconsistency (of effects among studies); imprecision, objectivity (that is, applicability of participants, interventions, and outcomes of the study question); and other modifying factors, including data dissemination (that is, sample size) and strength-of-effect estimates. By combining the item scores for each of these domains, we determined the level of evidence ( Table 4 ), which was classified into four categories:

**Table 4:** Quality of studies according to the GRADE system

Outcome; no. of participants (studies)	Relative effect (95%CI)	Anticipated absolute effects (95%CI)	Certainty
		Difference	
The use of rhinomanometry in mouth breathing assessed with rhinomanometry; no. of participants: 118 (3 observational studies)	Studies have shown that the use of rhinomanometry is reliable due to its respective analysis of respiratory function parameters and the existence of correlations regarding the variables of nasal permeability tests for the interdisciplinary treatment of pediatric patients with mouth breathing. However, among studies that evaluated the effectiveness of surgical treatments and procedures such as adenotonsillectomy and rapid maxillary expansion, the results were followed by slight changes in nasal resistance only to improve nasal function in subjects with breathing difficulties.		⨁◯◯◯ Very low 1 2 3 a b
*The **risk** in the **intervention group** (and its 95%CI) is based on the assumed risk in the comparison group and the **relative effect** of the intervention (and its 95%CI).			
**GRADE Working Group grades of evidence** **High certainty:** we are very confident that the true effect lies close to that of the estimate of the effect. **Moderate certainty:** we are moderately confident in the effect estimate: the true effect is likely to be close to the estimate of the effect, but there is a possibility that it is substantially different. **Low certainty:** our confidence in the effect estimate is limited: the true effect may be substantially different from the estimate of the effect. **Very low certainty:** we have very little confidence in the effect estimate: the true effect is likely to be substantially different from the estimate of the effect.			

**Abbreviations:** 95%CI, 95% confidence interval; GRADE, Grading of Recommendations Assessment, Development and Evaluation.

a There was heterogeneity, the studies differed regarding the type of intervention and the duration of the follow-up, and they presented unexplained statistically significant analysis of the sensitivity results (nasal area and nasal resistance of acoustic rhinometry and rhinomanometry before and after intervention).

b The sample size of the studies is small.

• High: there is high confidence that the true effect lies close to that of the estimate of the effect.
• Moderate: there is moderate confidence in the estimated effect.
• Low: confidence in the effect is limited.
• Very low: confidence in the effect estimate is very limited. There is an important degree of uncertainty in the findings.

One researcher assessed the GRADE evidence level for each systematic review, while a second researcher reviewed and verified the assessments; discrepancies were solved through discussion.

## Results

**Fig. 1:**
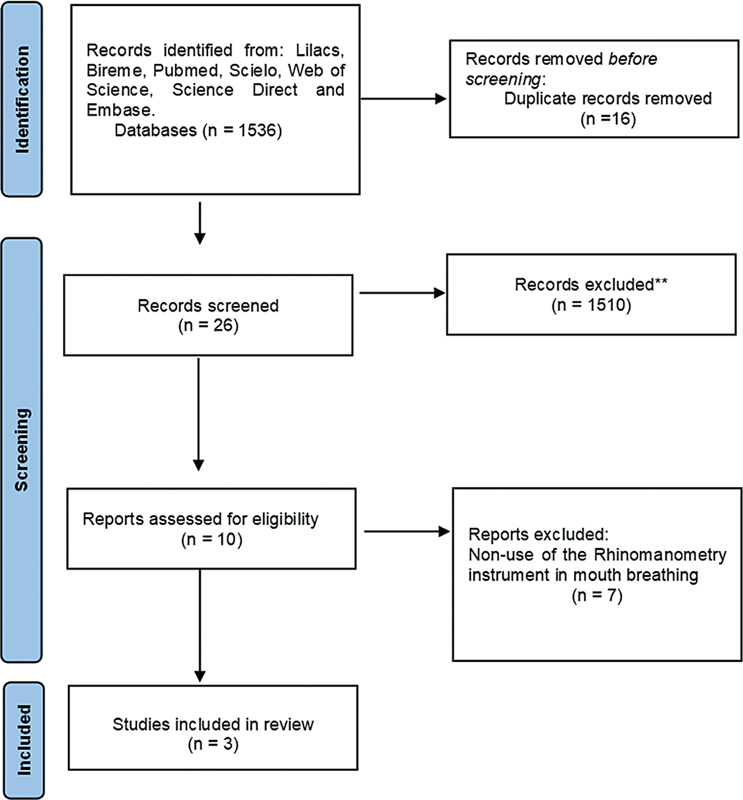
Flowchart of the selection of studies. Note: n = number of studies.

Initially, 1,536 articles were found, 1,510 of which were excluded after reading the title and abstract. Of the remaining 26 articles, 16 were duplicates, and 10 were selected for full-text reading, after which 6 were excluded, totaling 3 articles 14 15 16 included that reported the effectiveness of rhinomanometry as a diagnostic tool for mouth breathing and, thus, were eligible for review ( Figure 1 ). Data from the articles included are described in Table 3 . The methodological characteristics of these articles are shown in Table 4 .

## Discussion

Over the last 20 years, rhinomanometry has been widely accepted and studied in the assessment of nasal permeability in people with mouth breathing. 17 According to the present review of articles on the relationship between rhinomanometry and mouth breathing, most discussed nasal obstruction, but did not feature mouth breathing as their main focus.

In the present research, we found three articles that addressed the proposed subject. They presented different characteristics in terms of sample, objectives, and methodological procedures, as well as in terms of not describing clinical trials and case-control comparisons. Therefore, given this heterogeneity, wee could not perform a meta-analysis. In addition, another aspect that made the homogeneous analysis impossible was the different measurement methodology in the pre- and postintervention moments for aspects such as maxillary expansion, vasoconstrictor administration, and adenotonsillectomy.

We identified three systematic reviews 14 15 16 of very low quality, and methodological weaknesses consistent with the GRADE ratings assigned in the assessment of the quality of evidence were observed in the results.

The present study had the following limitations. First, the studies included differed in terms of intervention methods, duration of follow-up, and outcome variables. regarding the two cohort studies, one 14 used orthodontic intervention, while the other study 16 performed ENT surgery. Furthermore, in the follow-up of the intervention time they were inconsistent. In the third article, a cross-sectional study, the authors 15 correlated rhinomanometry with other objective methods, which varied in the outcomes. In addition, we observed the inaccuracy of the studies regarding sample size, as well as lack of representation of the unexposed group in the one of the cohort studies. 16 However, the rhinomanometry methodology used in the studies was reliable, as it analyzed the parameters of nasal respiratory function.

The clinical relevance of the findings depends on normal rhinomanometric values and the initial nasal resistance. The fact that normal values change with age 18 makes it difficult to establish the clinical relevance. Regarding nasal resistance, the normal value for adults is 0.3 Pa s/cm ^3^ , while for children, it is 0.4 Pa s/cm ^3^ . According to these values, a reduction of 0.12 Pa s/cm ^3^ is considered clinically relevant.

Since the rhinomanometry examination is considered the gold standard, because it is objective and has clear and standardized measurements, 19 the present review included, articles in which nasal function was evaluated through rhinomanometry to aid in the diagnosis of mouth breathing in pediatric patients. However, among the methods of the articles included, the following stand out: cephalometry, acoustic rhinometry, and computed tomography. These methods focus on anatomical changes but not on nasal function.

Currently, rhinomanometry is used as a diagnostic approach to mouth breathing, especially with the participation of an interdisciplinary team composed of dentists, otolaryngologists, and/or speech therapists, in order to obtain evidence of the functional analysis of the structures involved in respiratory function. Therefore, rhinomanometry is used in the field of otorhinolaryngology (ORL) to assess the effectiveness of treatments and surgical procedures, and dentists use it to investigate skeletal structures in orthodontic procedures. 20 21 Due to these facts, large gaps are found among the evaluated publications.

Regarding the type of study included in the present review, one was a cross-sectional quantitative study, 15 and the other two were cohort studies. 14 16 Another relevant aspect of the articles was the small sample, which showed a reduced representation in the first 15 and second articles, 14 with samples of 29 and 30 subjects respectively. In the third article, 16 there were 30 and 29 participants in each group. Thus, we assume that this reduced number of subjects may have compromised the reproducibility of the findings for the general population. 22

The population of the three studies consisted of children aged between 2 and 13 years. The choice of age group can be understood due to the fact that mouth breathing is common in children, and because there is has a higher frequency of mouth breathing in the pediatric and school-age public. 1 14 23 Likewise, the characteristics of mouth breathing in terms of lowered tongue posture and lengthening of the lower anterior facial height are evident at 3 years of age, but are more frequently detected after 5 years of age. The deleterious impact of decreased nasorespiratory function is practically complete in adolescence.

The objective assessment of nasal permeability is of great importance for a better understanding of nasal obstruction, as its objective data provide a comparison between clinical and surgical treatments. Therefore, there is a need to study nasal physiology more effectively. Currently, AAR is the most used method to assess nasal resistance, 19 which quantifies airflow and transnasal pressure over a given period. The flow is measured using a pneumotachograph, whose terminal is directly adjusted to the nasal cavity to be examined or connected to an appropriate mask. 24

In the temporal analysis, it was possible to find publications from the last eight years, thus indicating a slight increase in research on the repercussions of mouth breathing. As for the spatial distribution, the studies 14 15 16 were carried out in Brazil, in the states of São Paulo and Minas Gerais. This geographic data shows the interest of Brazilian researchers 23 in the use of rhinomanometry in the diagnosis and evaluation of mouth breathing.

Regarding the rhinomanometry devices, it is worth highlighting the predominance of a device manufactured in Denmark, the SR 2000 (Rhinometrics A/S, Smørum, Hovedstaden, Denmark) with nasal adapters, 14 16 while another study 15 used an equipment from Scotland, the A1/NR6 (GM Instruments Ltd., Kilwinning, Ayrshire, United Kingdom). This highlights the need for devices that perform objective tests to measure the nasal resistance of the upper airways (UAs).

In addition to these technological data, it is noteworthy that the rhinomanometry device is a sophisticated equipment, difficult to transport and dependent on technical assistance. 4 Another important point refers to the different models of computed AAR, especially the four-phase one, which represents the next generation of rhinomanometry and offers a better resolution for the analysis of breathing over time with new variables that correlate with other data from objective evaluations. 25 This four-phase rhinomanometry device requires attention, instrument hygiene, patient care, and proper positioning for optimal results.

Concerning the sample populations, attention is drawn to the predominant applicability in children with mouth breathing of different etiologies, in a cross-sectional study 15 with maxillary atresia and unilateral or bilateral posterior crossbite, and in another study, 14 with prospective cohort design, with patients with posterior crossbite. Finally, the third study 16 showed a distribution in two groups: one with mouth breathing and another with UA obstruction due to adenotonsillar hyperplasia (ATH).

In the cross-sectional study, 15 the authors performed a correlation regarding the methods of assessment of computed AAR, acoustic rhinometry, and cone-beam computed tomography in mouth breathers with transverse maxillary deficiency. The exams were performed before and after the administration of vasoconstrictor, and negative correlations were found, between: width of 4 and mean inspiratory resistance (Rho = -0.385); mean inspiratory resistance assessed before administration of vasoconstrictor and nasal volumes from 0 cm to 5 cm (Rho = -0.382); and mean expiratory resistance assessed before administration of vasoconstrictor and minimum cross-sectional areas 1 (Rho = -0.362). Correlations can be observed between rhinomanometry and other quantitative tests that play a role in measuring the effect of therapeutic interventions.

About the prospective cohort study, 14 the authors evaluated the effects of rapid maxillary expansion on the nasal cavity and on facial morphology through rhinomanometry and acoustic rhinometry. A significant increase in the bone width of the nasal cavity and in the maxilla was found, in addition to a slight decrease in nasal resistance without the use of nasal decongestion. Therefore, it is possible to consider that methods for nasal permeability quantification are important to understand orthodontic measures.

Regarding the cohort study, 16 changes in systolic pulmonary artery pressure (SPAP) and total inspiratory nasal flow (TINF) were evaluated by means of Doppler echocardiography and AAR before and after six months of adenoidectomy and/or tonsillectomy. The authors found that the mean values of the mouth-breathing group; in the preoperative period, while the mean nasal patency was of 42.85% (± 17.83%; *p* = 0.01), in the postoperative period, this value was of 79.33% (± 21.35; *p* = 0.01). However, they 16 found statistically significant differences between the mean values of the percentage of nasal patency before and after the surgical procedure ( *p* < 0.001). These findings indicate the effectiveness of adenoidectomy and/or tonsillectomy in improving nasal respiratory function in children with oral breathing.

Few studies have compared the cardiopulmonary alterations in children with mouth breathing 27 and even more rare are those that have analyzed such alterations (correlating them with the objective measurement of the nasal obstructive condition), as they may influence the clinical practice of specialists, with a change in the approach to mouth breathers, with warnings regarding risks not yet very well established. 28 Thus, it is suggested that research on the subject with control groups should be carried out to provide greater reliability to the results. Furthermore, for evidence-based practice, that is, for a decision to be taken professionally based on the scientific results obtained, it is ideal that such studies present a high strength of evidence through randomized clinical trials. 26

In general, we could observe that the effectiveness of the treatment varied according to the rhinomanometry applied in combination with different treatments, such as in orthodontic, surgical and drug procedures. However, the objective evaluation of the effectiveness of rhinomanometry in the diagnosis in oral breathing it still lacks standardization. 29

## Conclusion

In the present review, the included studies used rhinomanometry in their population of mouth breathers; however, there was a lack of standardization of this instrument to test the efficiency of nasal resistance in the UAs, as well as a methodological variability that decreased the reliability of the results found. Thus, it would be desirable to conduct more controlled studies in this field and with larger samples to obtain a better understanding of the consequences of mouth breathing on nasal permeability.

## References

[1] Cunha D A Krakauer L Manzi S HMB Frazão Y Mouth Breathing: Assessment and Speech Therapy São José dos Campos 2019 491 501

[2] Moraes B Bezerra T Nasal Obstruction and Mouth Breathing Syndrome 2019 483 489

[3] Sakano E Sarinho E Cruz A IV Brazilian Consensus on Rhinitis-2017: Saline solution In: Joint document of the Brazilian Association of Allergy and Immunology,Brazilian Association of Otorhinolaryngology and Cervico-Facial Surgery and Brazilian Society of Pediatrics.2017 3 43

[4] Roithmann R Philip C Objective Assessment of Nasal Patency: Why, When, How? Rev Bras Otorrinolaringol (Engl Ed) 1995 61 10 109

[5] Nathan R A Eccles R Howarth P H Steinsvåg S K Togias A Objective monitoring of nasal patency and nasal physiology in rhinitis J Allergy Clin Immunol 2005 115(3, Suppl 1)S442 S459 15746882 10.1016/j.jaci.2004.12.015 PMC7112320

[6] Mendes A Wandalsen G Solé D Objective and subjective methods of nasal obstruction assessment Rev Bras Alerg Imunopatol 2011 34 06 234 240

[7] Kaneda S Iida M Yamamoto H Evaluation of Nasal Airflow and Resistance: Computational Modeling for Experimental Measurements Tokai J Exp Clin Med 2019 44 03 59 67 31448398

[8] Giotakis A I Tomazic P V Riechelmann H Vent J Objective Assessment of Nasal Patency Facial Plast Surg 2017 33 04 378 387 28753711 10.1055/s-0037-1604356

[9] Chandra R K Patadia M O Raviv J Diagnosis of nasal airway obstruction Otolaryngol Clin North Am 2009 42 02 207 225, vii 19328887 10.1016/j.otc.2009.01.004

[10] Gulati S P Sachdeva O P Wadhera R Sodhi N Garg A Role of rhinomanometry to assess nasal airflow and resistance in patients undergoing septoplasty Indian J Otolaryngol Head Neck Surg 2008 60 02 133 136 23120521 10.1007/s12070-007-0119-x PMC3450507

[11] Wandalsen G F Mendes A I Solé D Correlation between nasal resistance and different acoustic rhinometry parameters in children and adolescents with and without allergic rhinitis Rev Bras Otorrinolaringol (Engl Ed) 2012 78 06 81 86 10.5935/1808-8694.20120038 PMC9446352 23306573

[12] Maher C G Sherrington C Herbert R D Moseley A M Elkins M Reliability of the PEDro scale for rating quality of randomized controlled trials Phys Ther 2003 83 08 713 721 12882612

[13] Brugnolli A Cavada L Saiani L Methodological guidelines: GRADE-Manual system for grading the quality of evidence and strength of recommendation for health decision-makingEditora MS. Ministério da Saúde. Secretaria de Ciência, Tecnologia e Insumos Estratégicos.2014:924–926.

[14] Itikawa C E Valera F CP Matsumoto M AN Lima W TA Effect of rapid maxillary expansion on the dimension of the nasal cavity and on facial morphology assessed by acoustic rhinometry and rhinomanometry Dental Press J Orthod 2012 17 04 129 133

[15] Sakai R HUS Marson F AL Sakuma E TI Ribeiro J D Sakano E Correlation between acoustic rhinometry, computed rhinomanometry and cone beam computed tomography in mouth breathers with maxillary atresia Rev Bras Otorrinolaringol (Engl Ed) 2018 84 01 40 50 10.1016/j.bjorl.2016.10.015 PMC9442894 28017262

[16] Ramos V M Nader C M Meira Z M Impact of adenotonsilectomy on nasal airflow and pulmonary blood pressure in mouth breathing children Int J Pediatr Otorhinolaryngol 2019 125 82 86 31271972 10.1016/j.ijporl.2019.06.025

[17] Ottaviano G Fokkens W J Measurements of nasal airflow and patency: a critical review with emphasis on the use of peak nasal inspiratory flow in daily practice Allergy 2016 71 02 162 174 26447365 10.1111/all.12778

[18] Zapletal A Chalupová J Nasal airflow and resistance measured by active anterior rhinomanometry in healthy children and adolescents Pediatr Pulmonol 2002 33 03 174 180 11836796 10.1002/ppul.10066

[19] Standardisation Committee on Objective Assessment of the Nasal Airway, IRS, and ERS Clement P AR Gordts F Consensus report on acoustic rhinometry and rhinomanometry Rhinology 2005 43 03 169 179 16218509

[20] Okuro R T Morcillo A M Ribeiro MÂ Sakano E Conti P B Ribeiro J D Mouth breathing and forward head posture: effects on respiratory biomechanics and exercise capacity in children J Bras Pneumol 2011 37 04 471 479 21881737 10.1590/s1806-37132011000400009

[21] Sahin-Yilmaz A Naclerio R M Anatomy and physiology of the upper airway Proc Am Thorac Soc 2011 8 01 31 39 21364219 10.1513/pats.201007-050RN

[22] Melo A C Gomes AdeO Cavalcanti A S Silva H J Acoustic rhinometry in mouth breathing patients: a systematic review Rev Bras Otorrinolaringol (Engl Ed) 2015 81 02 212 218 10.1016/j.bjorl.2014.12.007 PMC9449077 25618769

[23] Lima A CD Cunha D AD Albuquerque R C Costa R NA Silva H JD Sensory changes in mouth breathers: Systematic review based on the prisma method Rev Paul Pediatr 2019 37 01 97 103 30110113 10.1590/1984-0462/;2019;37;1;00012 PMC6362378

[24] Paiva J B Nasal cavity rhynometric study in patients submitted to rapid maxillary expansion Ortodontia 2000 ••• 36 42

[25] Vogt K Jalowayski A A Althaus W 4-Phase-Rhinomanometry (4PR)–basics and practice 2010 Rhinol Suppl 2010 21 1 50 20649107

[26] Clancy C M Slutsky J R Patton L T Evidence-based health care 2004: AHRQ moves research to translation and implementation Health Serv Res 2004 39 05 xv xxiii 15333128 10.1111/j.1475-6773.2004.00286.x PMC1361066

[27] Silveira Wd Mello F C Guimarães F S Menezes S L Postural alterations and pulmonary function of mouth-breathing children Braz J Otorhinolaryngol 2010 76 06 683 686 21180932 10.1590/S1808-86942010000600002 PMC9443736

[28] Pacheco M CT Casagrande C F Teixeira L P Guidelines proposal for clinical recognition of mouth breathing children Dental press journal of orthodontics 2015 39 44 26352843 10.1590/2176-9451.20.4.039-044.oar PMC4593528

[29] Sunnergren O Ahonen H Holmström M Active anterior rhinomanometry: A study on nasal airway resistance, paradoxical reactions to decongestion, and repeatability in healthy subjects Laryngoscope Investigative Otolaryngology 2023 8 05 1136 1145 37899860 10.1002/lio2.1157 PMC10601575

